# Social learning in a nocturnal marsupial: is it a possum-ability?

**DOI:** 10.1098/rsbl.2022.0460

**Published:** 2023-01-18

**Authors:** Emma J. Godfrey, Elissa Z. Cameron, Graham J. Hickling

**Affiliations:** ^1^ School of Biological Sciences, University of Canterbury, Private Bag 4800, Christchurch 8041, New Zealand; ^2^ Wildlife Ecology and Management, Manaaki Whenua Landcare Research, PO Box 69040, Lincoln 7640, New Zealand

**Keywords:** social learning, common brushtail possum, *Trichosurus vulpecula*, vertebrate pest, eradication

## Abstract

Social learning can reduce the costs associated with trial-and-error learning. There is speculation that social learning could contribute to trap and bait avoidance in invasive species like the common brushtail possum (*Trichosurus vulpecula*)—a marsupial for which social learning has not previously been investigated. In large outdoor pens, we presented wild-caught ‘demonstrator’ possums with puzzle devices containing an attractive food reward; 2 of 8 demonstrators accessed the reward the first night the puzzle was presented and another three succeeded on later nights. Meanwhile, ‘observer’ possums in adjacent pens watched the demonstrators for five nights and then were given the opportunity to solve the puzzle themselves; 15 of 15 succeeded on their first night (a highly significant improvement). This experiment thus provides strong evidence of social learning by common brushtail possums. Future research should investigate whether information about aversive stimuli (such as traps and toxic baits) can similarly be transmitted between possums by social learning; if so, this could have important implications for possum pest control.

## 1. Introduction

Learning through individual experience requires trial and error, which can be costly because of increased energy expenditure, vulnerability to predation and health consequences [1]. These costs could be reduced if individuals learnt by observing other individuals, thereby acquiring new knowledge with less risk (social learning) [2]. Social learning has been documented across a wide range of taxa including fishes, birds and eutherian mammals [2,3]. Spread of socially learned behaviours can occur rapidly over large geographical scales; for example, isolated incidents of sulfur-crested cockatoos (*Cacatua galerita*) opening household waste bins in three suburbs rapidly spread to 44 suburbs [4]. Similarly, historical data from early American whaler logbooks has revealed that naive sperm whales (*Physeter microcephalus*) socially learned defensive measures against whalers from more experienced whales, resulting in a rapid decline in harpooning success [5].

Although known to be widespread taxonomically, social learning is rarely explicitly examined in metatherian (marsupial) mammals [6]. In past learning studies, metatherians have typically been reported as being ‘behaviourally inferior’ [6] or ‘less evolutionary complex’ than eutherian mammals, despite the two sharing similar cognitive characteristics [7]. More recent studies have challenged these descriptions; for example, wild kangaroos (*Macropus fuliginosus fuliginosus*) actively gaze at experimenters during an unsolvable task, suggesting intentional human-directed communication [8]—a skill seen in eutherian mammals such as dogs, pigs and horses [9].

Metatherian learning studies mainly explore introduced predator recognition [10]. Rufous bettongs (*Aepyprymnus rufescens*) that learn to fear dogs can generalize this fear to foxes (*Vulpes vulpes*) [11]. Similarly, tammar wallabies (*Macropus eugenii*) that learn caution towards a model fox generalize this response to cats (*Felis catus*) [12]. Furthermore, predator-naive ‘observer’ wallabies displayed significantly higher vigilance levels towards a model fox after observing a ‘demonstrator’ wallaby that was fearful of the model than when the demonstrator was indifferent to the model, suggesting marsupial social learning abilities [3].

The common brushtail possum (*Trichosurus vulpecula*; hereafter ‘possum’) is a nocturnal Australian marsupial that, following their successful introduction in 1858, has become a major invasive pest in New Zealand (NZ) [13]. In Australia, possums typically occur at low densities (less than 1 possum ha^−1^; [14]) and are relatively solitary but in NZ, where there is abundant food and an absence of predators, densities can reach up to 16 possums ha^−1^ [14], increasing the potential for the social transmission of information. These over-abundant NZ populations are subjected to a variety of pest suppression measures, including traps and toxins [15,16] that can produce learned aversions ('bait shyness' or 'trap shyness') including conditioned taste aversions (CTAs) [17,18]. It is unknown, however, whether social learning contributes to these learned responses; if so, social learning could be contributing to the difficulty of eradicating possums from NZ [19].

In this study, we investigated the learning abilities of common brushtail possums through direct experience and social transmission. Specifically, we evaluated (i) whether demonstrator possums could learn to open specific targets on a puzzle device, and (ii) whether this learning could be socially transmitted to neighbouring observer possums.

## 2. Method

### (a) Animals

Wild-caught possums were transported to the Manaaki Whenua—Landcare Research (MWLR) captive animal facility at Lincoln, NZ where they were acclimated to captivity for two weeks before use in our experimental trials. Possums were individually housed in 3 × 4 × 3 m outdoor pens with mesh wire walls. Pens were arranged in rows with shared side walls, allowing possums in adjacent pens to see each other. Each pen contained branches or beams for climbing and a corrugated iron shelter with a hanging hessian sack for the possum to sleep in. Possums were fed daily on a maintenance diet of fruits and vegetables plus ad libitum cereal pellets and water.

### (b) General procedure

The experiment used two cohorts of healthy adult possums, each weighing 1.8 kg or more. Cohort 1 (tested in May 2021) consisted of 18 possums: six ‘demonstrators’ (four females and two males) that had to solve a puzzle through trial and error, and 12 ‘observers’ (all male) that were able to watch a demonstrator learning to solve its puzzle before being given an opportunity to solve it themselves. Cohort 2 consisted of two demonstrators (both female) and four observers (all males) that were in a second trial run with slightly different methods in November–December 2021 [20].

Observers were housed in pens on either side of a central pen containing their demonstrator; these triads of possums (electronic supplementary material, figure S1) produced a 2 : 1 learning ratio for the duration of the experiment.

### (c) Learning phase

**Figure 1.**
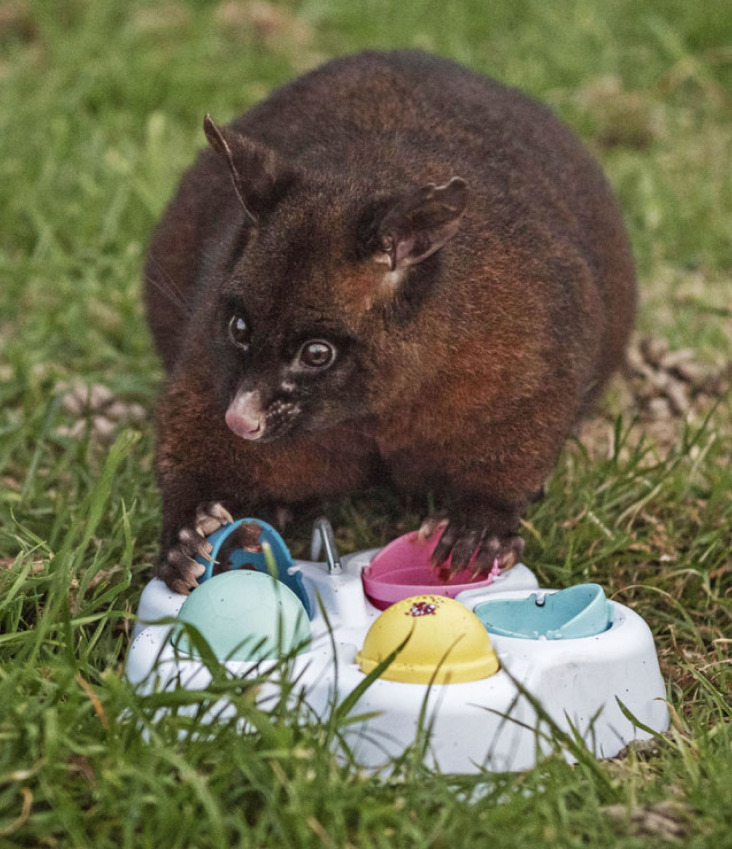
A possum interacts with the plastic puzzle device consisting of different coloured domes.

All individuals were initially naive to the puzzle and had not seen other possums interacting with it. Cohort 1's learning phase involved adding a plastic puzzle toy (figure 1) to each demonstrator's pen for five nights, during which time the demonstrator could physically interact with it, while observers in the two adjacent pens could only watch. The puzzle, secured to the ground using tent pegs, consisted of five differentially coloured domes that could be manually flipped open. The night before the learning phase began, an empty puzzle with shut dome lids was placed in the pen of each demonstrator so that the possum would not be frightened by it on the night the learning trial began. For the next five nights, food rewards (2 g of chocolate hazelnut spread in each) were placed inside the pink and yellow ‘target’ domes, both of which were marked with a dot of sparkling nail polish on the top. In the remaining ‘non-target’ domes (green, light blue and dark blue), spread was placed underneath a layer of masking tape and duct tape, so those domes contained the scent of the spread without it being easily accessible to the possum. The puzzles were refreshed daily by returning them to their original location if moved and replenishing the hazelnut spread within each dome.

The same procedure was used for cohort 2 possums, which participated in a slightly different trial (statistical justification for their use in these analyses is provided as the electronic supplementary material), except the light blue and dark blue domes contained chocolate hazelnut spread mixed with 5% by weight denatonium benzoate—food reward was added to the yellow and pink domes, as in the previous trial. The denatonium benzoate additive was intended to be an aversive treatment but was found to be non-aversive and unattractive (see [20]). Consequently, for cohort 2 we considered the two blue domes to be functionally equivalent to the empty green dome.

To record interactions with the puzzle, we used motion-detecting cameras with infrared illuminators (Bushnell Trophy Cams and similar models) to record time-stamped video clips of each possum's night-time activity. Two cameras were placed in each demonstrator's pen: one directly above the puzzle facing downwards and the other at ground level, facing the shelter and puzzle (electronic supplementary material, figure S1). This positioning allowed the cameras to record when the possum first left its shelter, first approached the puzzle and any subsequent interactions with the puzzle including the opening of domes. Concurrently, a single camera was placed in each observer's pen to record when it emerged from its shelter; this allowed us to determine whether these possums had the opportunity to observe their demonstrator while it was interacting with its puzzle.

### (d) Testing for social learning

To test whether observers had learnt by watching their demonstrator solve its puzzle, during this phase we removed each demonstrator's puzzle and instead placed a puzzle with food rewards in each observer's pen for five nights. Each observer possum received a new, clean puzzle—rather than a reused puzzle from a demonstrator—to ensure leftover scent cues from the demonstrator did not affect their learning. The puzzles were set up as before (i.e. food rewards in the pink and yellow ‘target’ domes) and activity was again recorded using two cameras.

### (e) Interaction terminology

Interactions with the puzzles were classified from 12 hours of video data each night, beginning when each possum first emerged from its sleep sack. Possums' interactions with the puzzle domes were scored as ‘deliberate’, ‘accidental’ or ‘did not open’ (table 1).

**Table 1.** Definitions of the terminology used to describe the possums' interactions with the puzzles and measures of activity during the experiment.

interaction term or measure	definition
approach	the possum moves close enough to the puzzle to be able to touch the domes with its snout or paws
time between approaching the puzzle and solving one target	time (measured in seconds) between the possum's first approach to the puzzle and their first opening (when the dome is moved in a way that exposes the inside) of a designated target
*openings*	
deliberate	the possum intentionally opens a dome (by using its snout or paws). Intentionality was inferred by the direct gaze of the possum towards the dome it was manipulating
accidental	the possum unintentionally opens a dome (by stepping on the dome)
did not open	the possum does not open any domes

### (f) Analysis

Data from cohorts 1 and 2 were pooled for analysis. The proportions of demonstrators and observers opening a target dome on the first night were compared using Fisher's exact test. The time it took demonstrators and observers to successfully open their first target dome was estimated by assuming 720 min of exposure if a possum failed to open a target on a given night (and compared using a Mann–Whitney *U*-test).

## 3. Results

### (a) Learning phase

On their first night's encounter with the puzzle, only 2 of the 8 demonstrators (25%) solved the puzzle (i.e. deliberately opened at least one target dome). Two more succeeded on night 2, and a fifth succeeded on night 4. Cumulative exposure to the puzzle before success ranged from 0.8 to 2121 min for the five successful possums (median = 720 min). Three of the demonstrators never solved the puzzle.

Of the four demonstrators that first solved the puzzle on night 1 or 2, each solved their puzzle faster on the next night for which we had data, which suggests the demonstrators were learning by trial and error.

### (b) Social learning phase

One of the 16 observer possums fell ill and was removed from the study and cameras failed to record three observers' first successful opening of a target dome; sample sizes have been adjusted accordingly.

**Figure 2.**
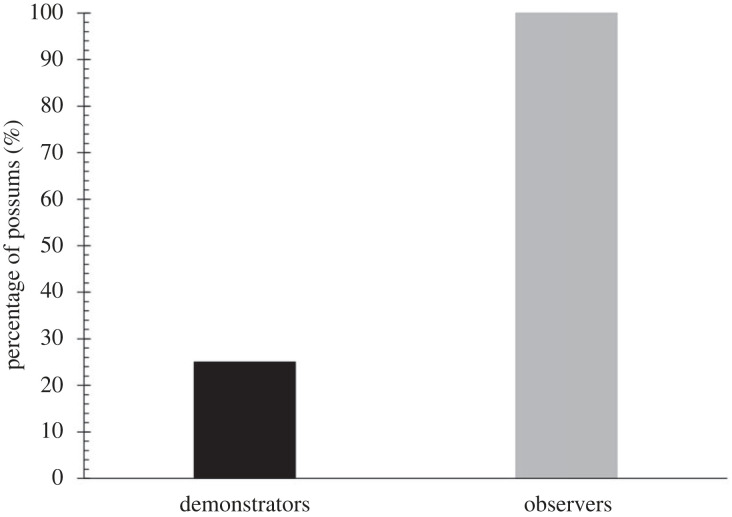
The percentage of possums to open a target dome on their first night of interaction with the puzzle, comparing demonstrators (dark bars, *n* = 8) to observers (light bars, *n* = 15).

All 15 healthy observers deliberately opened at least one target dome on their first night of interaction with the puzzle; this 100% success rate was much higher than the demonstrators had achieved (25%; figure 2; Fisher's exact test, *p* < 0.01).

Cameras recorded 12 of the 15 observers opening their first target dome; 7 of the 12 did so within 1 min of approaching the puzzle and 10 did so within 3 min. The other two observers took much longer (71 min and 177 min, respectively). Although there was a large variation in time-to-success in both groups, the observers were nevertheless much faster overall in solving the puzzle than were the five successful demonstrators (median times of 0.7 min and 720.5 min, respectively; Mann–Whitney *U* = 7.5, *p* < 0.05). Overall, only 3 of the 8 (38%) demonstrators deliberately opened the target whereas 14 of the 15 (93%) observers did the same.

## 4. Discussion

To our knowledge, these findings are the first documented evidence of social learning in common brushtail possums. There was a high variability in the demonstrators' time-to-success as they learned through trial and error over multiple nights. Some demonstrators never learnt to open a target, increasing this variability. Conversely, the observers’ responses to the puzzle were much faster and much less variable, suggesting they learned how to interact with the puzzle by watching the demonstrators.

Half of the demonstrators from cohort 1 never opened a target during their testing, so we ran an additional night post-trials to see if these unsuccessful demonstrators had subsequently learnt from their observers who successfully solved the puzzle. Two of the three succeeded when retested, again suggesting social learning—the final possum never showed interest in the puzzle.

Although wild possums are relatively solitary, they nevertheless have extensively overlapping home ranges, particularly at high densities and are often attracted to localized resources such as fruit trees or baiting stations, enhancing the potential for the social transmission of information. Close interactions between males commonly only occur during the mating season [21] while females have close interactions with others of both sexes year-round [22]. In our pens, possums had the opportunity to watch neighbours that were in proximity, enhancing opportunities for the social transmission of information.

Some past studies have suggested that the sex of the demonstrator relative to the observer can affect social learning (e.g. [23,24]). Only male observers and mainly female demonstrators were made available to us for this experiment, so potential differences in the social transmission of learning between the sexes could not be fully explored. We saw no obvious differences in interactions with the puzzle (i.e. latency to approach and time to solving one target) by male observers that watched a female demonstrator relative to those who watched another male (see [20]), but more research on this question is needed, as it has been suggested that female demonstrator-female observer pairings may be fast social learners (see [20]).

Previous possum learning studies have focused on inducing and/or overcoming CTAs—CTAs involve different neurophysiological pathways than most other kinds of learning [25]. Our experiment differed from traditional CTA studies in that it used an attractive, positive, taste reward to stimulate the possums' learning. Furthermore, CTAs are linked to taste sensory input, whereas our experiment incorporated visual inputs from the puzzle, differing from past studies. Studies from the 1970s (mentioned in [26]) have suggested possums have difficulty in visual-based discrimination tasks but this conclusion has since been challenged (e.g. [26,27]).

The observer possums may have learned to associate the demonstrators’ food rewards with the colour of the target dome; however, the importance of colour as a cue is unclear as little is known about colour vision in common brushtail possums. Vlahos [28] proposed that possums' perception of colours is based on dichromatic colour vision but later reported finding a potential third cone opsin population comprised a small number of unlabelled cones [29] which was suggestive of trichromatic colour abilities. This has yet to be confirmed with behavioural evidence [29]. Cone-mediated colour vision would be most effective at high light levels—our possums all emerged during twilight, which may have provided a window of opportunity to interact with the puzzle while able to perceive the domes’ colours.

Our research provides, to our knowledge, the first evidence of social learning capabilities in common brushtail possums. These social learning abilities may help explain why some recalcitrant individuals in NZ populations are difficult to eradicate. We urge caution on this point, however, until there is evidence possums' social learning of a positive stimulus can indeed be generalized to social learning of negative encounters, for example, sub-lethal interactions with traps and toxins. This question should be a priority for future research because of its relevance to NZ's current Predator Free initiative.

## Data Availability

Data available from the Dryad Digital Repository: https://doi.org/10.5061/dryad.h44j0zpps [30]. The data are provided in the electronic supplementary material [31].
